# Site staff perspectives on communicating trial results to participants: Cost and feasibility results from the Show RESPECT cluster randomised, factorial, mixed-methods trial

**DOI:** 10.1177/17407745231186088

**Published:** 2023-07-29

**Authors:** Annabelle South, Julia Bailey, Barbara E Bierer, Eva Burnett, William J Cragg, Carlos Diaz-Montana, Katie Gillies, Talia Isaacs, Nalinie Joharatnam-Hogan, Claire Snowdon, Matthew R Sydes, Andrew J Copas

**Affiliations:** 1 MRC CTU at UCL, Institute of Clinical Trials and Methodology, University College London, London, UK; 2 Department of Primary Care and Population Health, University College London, London, UK; 3 Brigham and Women’s Hospital, Harvard Medical School, Boston, MA, USA; 4 Clinical Trials Research Unit, Leeds Institute of Clinical Trials Research, University of Leeds, Leeds, UK; 5 Health Services Research Unit, University of Aberdeen, Aberdeen, UK; 6 UCL Institute of Education, University College London, London, UK; 7 London School of Hygiene & Tropical Medicine, London, UK

**Keywords:** Feedback of results, communicating results, researcher perspective, researcher–participant relations, trial conduct, trial ethics, mixed methods

## Abstract

**Background/Aims:**

Sharing trial results with participants is an ethical imperative but often does not happen. Show RESPECT (ISRCTN96189403) tested ways of sharing results with participants in an ovarian cancer trial (ISRCTN10356387). Sharing results via a printed summary improved patient satisfaction. Little is known about staff experience and the costs of communicating results with participants. We report the costs of communication approaches used in Show RESPECT and the views of site staff on these approaches.

**Methods:**

We allocated 43 hospitals (sites) to share results with trial participants through one of eight intervention combinations (2 × 2 × 2 factorial; enhanced versus basic webpage, printed summary versus no printed summary, email list invitation versus no invitation). Questionnaires elicited data from staff involved in sharing results. Open- and closed-ended questions covered resources used to share results and site staff perspectives on the approaches used. Semi-structured interviews were conducted. Interview and free-text data were analysed thematically. The mean additional site costs per participant from each intervention were estimated jointly as main effects by linear regression.

**Results:**

We received questionnaires from 68 staff from 41 sites and interviewed 11 site staff. Sites allocated to the printed summary had mean total site costs of sharing results £13.71/patient higher (95% confidence interval (CI): −3.19, 30.60; p = 0.108) than sites allocated no printed summary. Sites allocated to the enhanced webpage had mean total site costs £1.91/patient higher (95% CI: −14, 18.74; p = 0.819) than sites allocated to the basic webpage. Sites allocated to the email list had costs £2.87/patient lower (95% CI: −19.70, 13.95; p = 0.731) than sites allocated to no email list. Most of these costs were staff time for mailing information and handling patients’ queries. Most site staff reported no concerns about how they had shared results (88%) and no challenges (76%). Most (83%) found it easy to answer queries from patients about the results and thought the way they were allocated to share results with participants would be an acceptable standard approach (76%), with 79% saying they would follow the same approach for future trials. There were no significant effects of the randomised interventions on these outcomes. Site staff emphasised the importance of preparing patients to receive the results, including giving opt-in/opt-out options, and the need to offer further support, particularly if the results could confuse or distress some patients.

**Conclusions:**

Adding a printed summary to a webpage (which significantly improved participant satisfaction) may increase costs to sites by ~£14/patient, which is modest in relation to the cost of trials. The Show RESPECT communication interventions were feasible to implement. This information could help future trials ensure they have sufficient resources to share results with participants.

## Background/aims

Offering trial participants the results of trials they have participated in is an important part of the ethical conduct of clinical trials.^1,2^ There is evidence from a broad range of studies that most trial participants want to receive trial results.^3–7^ However, in practice, it often does not happen or is done poorly. The 2021 UK Health Research Authority research transparency report states that ‘90% of clinical trials have not told participants about findings’.^8^ A 2016 survey of authors of clinical trials publications from 2014 to 2015 found that only 27% of them reported disseminating results to participants.^9^

Barriers to communicating results include practical challenges; concern about the impact of sharing results; uncertainty about how to do it; lack of guidance or incentives^9,10^ or researchers simply not thinking about it.^11^ Cost is a major barrier to sharing results with participants, with many trials not having budgeted for this activity.^9,12–16^ Sharing of results often occurs after the trial funding period, making it difficult to cover these costs. Trial staff may also have moved to work on new projects, leaving insufficient human resources for this activity.^9,15–17^

The literature on the feasibility and resource requirements for different approaches to sharing results with trial participants is sparse. The US Center for Information and Study on Clinical Research Participation reported that, when results were returned, each site spent around 30 min to 2 h sending out result summaries to trial participants^18^; although the number of participants at these sites was not discussed. Another study reported the costs of £1624 for running an online meeting for participants and other stakeholders.^19^ However, this cost estimate does not include the 40 h of staff time required to organise the event. The other cost estimate reported in the literature is from a study which sent out leaflets by post, with printing and postage cost coming to £1.22 per participant, excluding the leaflet development and mailing time.^5^ Better information on what are the resource requirements for approaches to sharing results could help researchers plan appropriately.

The Show RESPECT study elucidated how to share trial results with participants, testing three approaches in a cluster randomised factorial trial,^7,20^ within the context of the ICON8 ovarian cancer trial.^21^ Data collected from trial participants showed that mailing printed summaries, alongside a link to a webpage ± email list, resulted in higher satisfaction with how the results were shared than the link to a webpage ± email list without the printed summary.^7,20^ Full results from the Show RESPECT study have been published as part of a doctoral thesis.^20^ This article aims to explore the resource implications and views of site staff on the acceptability and feasibility of these approaches to sharing results.

## Methods

We used a mixed-methods approach to collect data from site staff on the resources required for sharing results (reported at cluster (site) level) and their views on the process, concerns and challenges faced (reported at individual level). The qualitative and quantitative data have equal weight in their contribution to addressing the research aims. For clarity, we refer to ICON8 participants as ‘patients’ and site staff who contributed data to Show RESPECT as ‘site staff’. The study protocol is available online.^22^

### Quantitative methods

Supplemental Table S1 contains the Consolidated Standards of Reporting Trials (CONSORT) 2010 checklist.

#### Trial design

Show RESPECT was a cluster randomised 2 × 2 × 2 factorial trial within the ICON8 ovarian cancer trial (ISRCTN10356387).^21^ We randomised each UK trial site (secondary or tertiary hospital) (cluster) in ICON8 that agreed to take part in Show RESPECT to a combination of interventions to communicate ICON8 results to participants.^7^ Sites were stratified for randomisation by the number of ICON8 patients alive at the time the results were available (small < 6, medium 6–11 and large ≥ 12). The primary outcome for the study overall, reported previously, was patient satisfaction with how the results were shared.^7,20^ This article reports results around site staff perspectives on communicating results to participants.

#### Interventions

Staff at participating sites mailed a Patient Update Information Sheet to all ICON8 patients who were known to be still alive informing them that trial results were available together with access information, including the URL of their randomised webpage (basic or enhanced) and email list sign-up instructions if randomised to the email list. The Patient Update Information Sheet told patients at sites randomised to the printed summary that they would be mailed a printed summary of the results after 3 weeks and how to opt out of receiving it. A full description of the interventions is contained in our previous report.^7,20^

#### Participants

For the results presented in this report, participants were site staff who had been involved in sharing the ICON8 results with people taking part in ICON8 (e.g. through mailing information or answering queries).

#### Outcomes

The primary outcome for this sub-study of Show RESPECT was the cost to site per participant. This is a composite endpoint made up of

- An estimate of the cost of the time taken to deliver the interventions at a site (multiplying the reported time taken by the cost per hour for the job role of the staff member(s) who delivered the interventions taken from the National Institute of Health Research Schedule of Events Cost Attribution Template, assuming a medical research charity funder);
- An estimate of the cost of the time taken to deal with queries;
- Any non-staff costs associated with result dissemination incurred by sites.

These costs were divided by the number of ICON8 patients at the site who were alive at the time results were shared.

Secondary outcomes from site staff data are listed in the S2 Text of the Supplemental Material.

Quantitative data were elicited from site staff by one questionnaire completed immediately after sharing results and another completed 2–3 months later (S3 Text of the Supplemental Material). Site staff involved in sending out printed information to patients were asked to complete the first questionnaire, and site staff involved in dealing with patient queries were asked to complete the second questionnaire. For some sites, this meant both questionnaires were completed by the same people, and for other sites, different people completed the two questionnaires. Data were collected from December 2018 to September 2019. All site staff involved in sharing results were asked to complete questionnaires while recording the time they (individually) spent on this. Where more than one member of staff returned questionnaires, the time (and costs, estimated based on their job role) was added together to estimate the total time/cost for that site.

The trial ended 4 months after the last batch of clusters was randomised, as further data were unlikely.

#### Sample size

Show RESPECT was powered based on the primary outcome (patient satisfaction).^7^ No sample size calculations were carried out to assess power for outcomes collected from site staff.

#### Randomisation

Sites in Show RESPECT were cluster randomised using a factorial approach to a combination of enhanced versus basic webpage; printed summary versus no printed summary; email list invitation versus no invitation. Sites were randomised in blocks of eight through random permutations within blocks. Full details of our randomisation approach can be found in our previous report.^7^

#### Blinding

It was not feasible to blind site staff to their site’s intervention allocation.

#### Statistical analysis

This analysis was conducted under the intention to treat (ITT) principle. We analysed data from site staff data according to the interventions their cluster was allocated to. The prior assumption in the trial design was that there would not be any important interactions between the webpages, printed summary and email list. Therefore, our analysis is of the main effects of each intervention, adjusting for the others in the regression models (alpha = 0.05). To reflect the way in which randomisation was carried out, we adjusted for site size stratum by including this as a variable in the regression models. We did not impute any missing outcomes, as we had no information to inform our imputation.

We estimated effect measures for the interventions based on regression models, using linear regression to analyse the costs of the interventions, ordinal logistic regression for ordinal outcomes and logistic regression for binary outcomes. Models include random effects for site. We used mean differences to summarise the effects for continuous outcome measures and odds ratios for binary and ordinal outcomes. As the population for this analysis is small, no subgroup analyses were performed.

Statistical analyses were performed using Stata version 16.1 (StataCorp LLC, College Station, TX).

### Qualitative methods

Supplemental Table S4 shows the Consolidated Criteria for Reporting Qualitative Studies (COREQ) checklist.

#### Qualitative sampling

Invitations to take part in interviews were sent by email to site staff involved in Show RESPECT. Purposive sampling included staff from sites offered the range of Show RESPECT interventions and different job roles (nursing, oncologist and administrative roles).

#### Qualitative data collection

Semi-structured interviews with site staff were carried out either face-to-face (on-site) or by telephone by A.S. S5 Text of the Supplemental Material contains more details about the research team and reflexivity. Semi-structured interviews were informed by a topic guide (S6 Text of the Supplemental Material), which was revised as interviews proceeded to explore emergent issues.^23^ Only the interviewee and interviewer were present during interviews. The interviews were audio recorded and transcribed verbatim. Field notes were made immediately afterwards. Transcripts were checked against the recordings for accuracy, and identifying data were redacted. Transcripts were not returned to interviewees for checking. Repeat interviews were not conducted. Free-text questions within the questionnaires invited comments alongside the quantitative data.

#### Qualitative analysis

A thematic analysis^24^ of qualitative data, taking a critical realist stance, was conducted by A.S. in Atlas.ti version 8.4 (ATLAS.ti Scientific Software Development GmbH), as detailed previously.^7^ Inductive thematic saturation was reached at the 11^th^ interview, as was data saturation. Meetings were held with site staff once the initial analysis had been carried out to check our interpretation and messages (rather than for data collection). A ‘following the thread’ approach was used to triangulate the results of the qualitative and quantitative components of the study at the analysis stage: The initial analysis of the two types of data was carried out to identify key themes and questions, then the other type of data was examined to see what light it could shed on those themes and questions.^25^

### Ethics

Show RESPECT obtained ethics approval from the London-Chelsea Research Ethics Committee, MREC number 18/LO/1011. Both questionnaires contained an embedded informed consent element, in line with the UK Health Research Authority’s guidance on proportionate approaches to informed consent for self-administered questionnaire-based research,^26^ with completion and return of the questionnaire taken to indicate consent to use the data has been given. Qualitative interviewees gave written informed consent before the interview started.

## Results

### Participation in Show RESPECT

**Figure 1.**
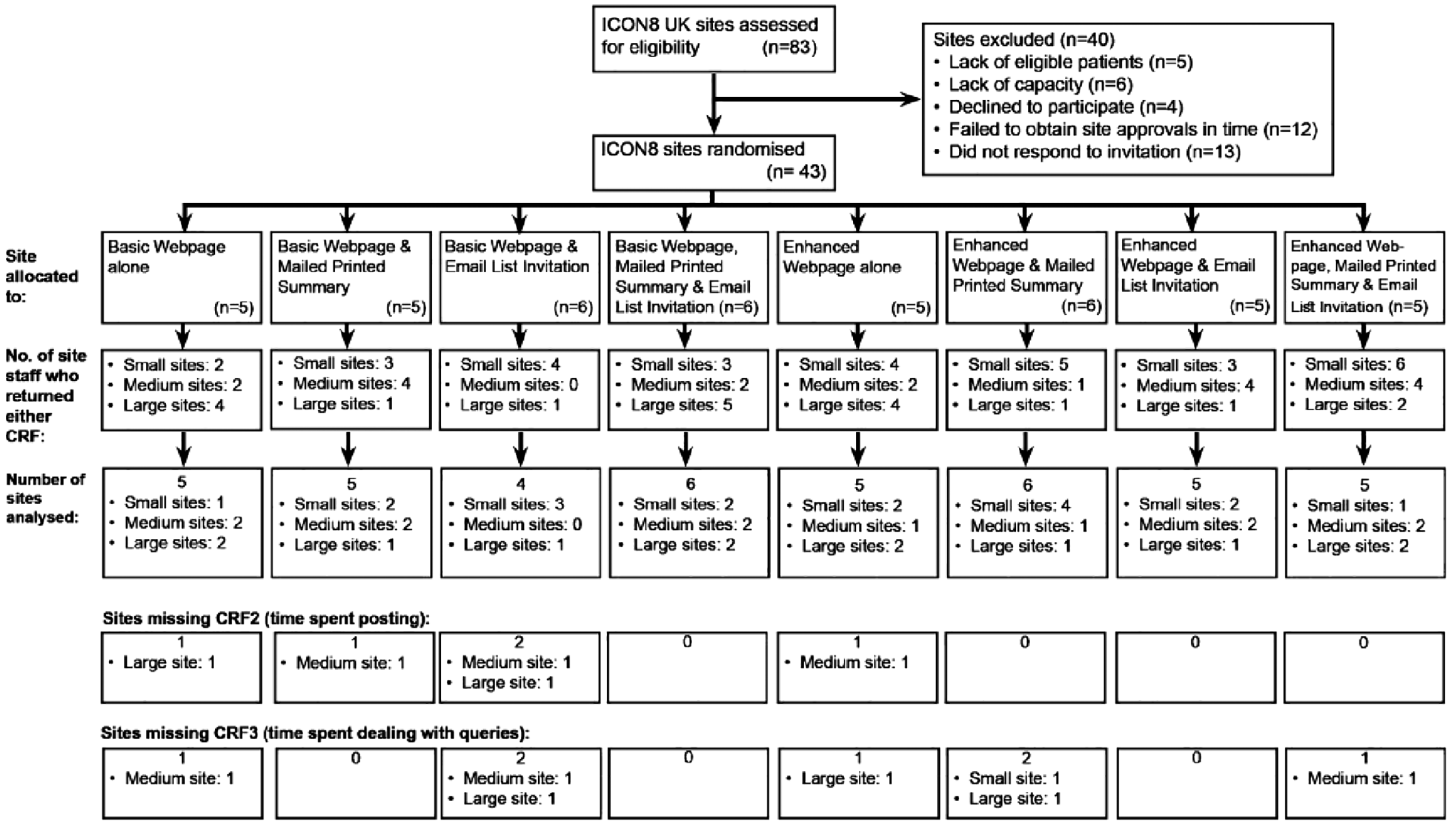
CONSORT diagram.

Show RESPECT randomised 43 sites. We received at least one questionnaire from 68 staff members from 41 sites. No questionnaires were returned from two randomised sites due to staff turnover. Figure 1 shows the CONSORT diagram, including the number of sites where no responses were received for the two site staff questionnaires by each of the eight combinations of interventions. Table 1 shows the number of sites and questionnaires received by the three factorial randomisations.

**Table 1.** Recruitment of sites and site staff respondents.

	Overall	Webpage		Printed summary		Email list	
	n (%)	Basic webpage, n (%)	Enhanced webpage, n (%)	No printed summary, n (%)	Printed summary, n (%)	No invitation, n (%)	Invitation, n (%)
Number of sites							
Total	43 (100)	22	21	21	22	21	22
Small sites^a^	17 (40)	8 (36)	9 (43)	8 (38)	9 (41)	9 (43)	8 (36)
Medium sites^a^	13 (30)	7 (32)	6 (29)	6 (29)	7 (32)	6 (29)	7 (32)
Large sites^a^	13 (30)	7 (32)	6 (29)	7 (33)	6 (27)	6 (29)	7 (32)
Number of site staff who returned either Case Report Form (CRF)							
Total	68	35	33	33	35	35	33
Small sites	28 (41)	14 (40)	14 (42)	13 (39)	15 (43)	14 (40)	14 (42)
Medium sites	19 (28)	8 (23)	11 (33)	8 (24)	11 (31)	9 (26)	10 (30)
Large sites	21 (31)	13 (37)	8 (24)	12 (36)	9 (26)	12 (34)	9 (27)
Minimum number^b^ of sites missing CRF2s (as % of sites) – time spent posting information							
Total	5 (12)	5 (23)	0 (0)	4 (19)	1 (5)	3 (14)	2 (9)
Small sites	0 (0)	0 (0)	0 (0)	0 (0)	0 (0)	0 (0)	0 (0)
Medium sites	3 (23)	3 (43)	0 (0)	2 (33)	1 (14)	2 (33)	1 (14)
Large sites	2 (15)	2 (29)	0 (0)	2 (29)	0 (0)	1 (17)	1 (14)
Minimum number^c^ of sites missing CRF3s (as % of sites) – time spent answering queries							
Total	6 (14)	3 (14)	3 (14)	4 (19)	2 (9)	3 (14)	3 (14)
Small sites	0 (0)	0 (0)	0 (0)	0 (0)	0 (0)	0 (0)	0 (0)
Medium sites	3 (23)	2 (29)	1 (17)	2 (33)	1 (14)	1 (17)	2 (29)
Large sites	3 (23)	1 (14)	2 (33)	2 (29)	1 (17)	2 (33)	1 (14)
Number of sites missing both CRFs (as % of sites)							
Total	2 (5)	2 (9)	0 (0)	2 (10)	0 (0)	0 (0)	2 (9)
Small sites	0 (0)	0 (0)	0 (0)	0 (0)	0 (0)	0 (0)	0 (0)
Medium sites	1 (8)	1 (14)	0 (0)	1 (17)	0 (0)	0 (0)	1 (14)
Large sites	1 (8)	1 (14)	0 (0)	1 (14)	0 (0)	0 (0)	1 (14)

a Sites were classified as small if they had fewer than 6 ICON8 participants alive at the time results were available, medium if they had 6–11 participants and large if they had 12 or more participants.

b Number of sites for which we had no responses (at least one response was expected from each site).

c Number of sites for which we had no responses, who had not confirmed that they received no queries from participants (CRF3 was only expected for sites that had received queries from participants).

Most respondents were in nursing (63%, 42) or administrative (30%, 20) roles. Most respondents worked on multiple trials simultaneously, with 72% (49) working on more than 10 trials at the time they completed the questionnaire. Around half (33) had been working on ICON8 for 2 years or less, while 38% (26) had worked on ICON8 for more than 5 years. Table 2 shows site staff respondent characteristics.

**Table 2.** Respondent characteristics.

Variable	Overall	Webpage		Printed summary		Email list	
	n (%)	Basic webpage, n (%)	Enhanced webpage, n (%)	No printed summary, n (%)	Printed summary, n (%)	No invitation, n (%)	Invitation, n (%)
Job role							
Medical	5 (7)	3 (9)	2 (6)	3 (9)	2 (6)	3 (9)	2 (6)
Nursing	42 (63)	23 (66)	19 (59)	22 (69)	20 (57)	23 (66)	19 (59)
Administrative	20 (30)	9 (26)	11 (34)	7 (22)	13 (37)	9 (26)	11 (34)
Years of experience working in trials							
Less than 1 year	9 (13)	4 (11)	5 (15)	4 (12)	5 (14)	4 (11)	5 (15)
1–5 years	24 (35)	11 (31)	13 (39)	14 (42)	10 (29)	12 (34)	12 (36)
6–10 years	16 (24)	9 (26)	7 (21)	6 (18)	10 (29)	9 (26)	7 (21)
More than 10 years	19 (28)	11 (31)	8 (24)	9 (27)	10 (29)	10 (39)	9 (27)
Number of trials they currently work on							
1–5	7 (10)	5 (14)	2 (6)	3 (9)	4 (11)	3 (9)	4 (12)
6–10	12 (18)	6 (17)	6 (18)	7 (21)	5 (14)	4 (11)	8 (24)
More than 10	49 (72)	24 (69)	25 (76)	23 (70)	26 (74)	28 (80)	21 (64)
Time currently spent working on ICON8							
Almost none	45 (67)	21 (62)	24 (73)	21 (66)	24 (69)	19 (56)	26 (79)
Around one day per week	21 (31)	13 (38)	8 (24)	10 (31)	11 (31)	14 (41)	7 (21)
Around two days per week	1 (1)	0 (0)	1 (3)	1 (3)	0 (0)	1 (3)	0 (0)
Number of years they have worked on ICON8							
Less than 1 year	16 (24)	6 (17)	10 (30)	9 (27)	7 (20)	6 (17)	10 (30)
1–2 years	17 (25)	10 (29)	7 (21)	12 (36)	5 (14)	12 (34)	5 (15)
3–4 years	9 (13)	3 (9)	6 (18)	5 (15)	4 (11)	5 (14)	4 (12)
5 years or more	26 (38)	16 (46)	10 (30)	7 (21)	19 (54)	12 (34)	14 (42)
Involvement in sharing the ICON8 results							
Sending information by post	55 (81)	26 (74)	29 (88)	28 (85)	27 (77)	31 (89)	24 (73)
Answering patient queries	43 (63)	24 (69)	19 (58)	23 (70)	20 (57)	18 (51)	25 (76)
Other	7 (10)	3 (9)	4 (12)	3 (9)	4 (11)	2 (6)	5 (15)

More than one respondent per site was allowed if more than one member of staff was involved in sharing results with participants.

We interviewed 11 site staff from 12 sites (mean duration 52 min, range: 35–75 min): two from small strata sites, five from medium strata sites and five from large strata sites. Most interviewees were in nursing (5) or administrative (4) roles, while two were oncologists. Table 3 shows the characteristics of interviewees. We recruited at least the target number of interviewees in all parts of the sampling frame.

**Table 3.** Site staff who were interviewed for the qualitative study.

Characteristics	No. of interviewees
Total interviewed	11
Show RESPECT randomisation^a^	
Works at site randomised to printed summaries	6
Works at site not randomised to printed summaries	6
Site strata (based on number of ICON8 participants)^a^	
Small	2
Medium	5
Large	5
Staff job role	
Oncologist	2
Nursing	5
Administrative	4

a One interviewee worked at two sites of different sizes, which were randomised to different interventions.

### Resources required from sites for sharing results with patients

The resources required from sites to share the results with patients include staff time for posting information and dealing with patient queries and the costs of postage and stationery (printed copies of materials were provided by the clinical trials unit). Table 4 shows the estimated total costs per patient and a breakdown by the different types of resources required. Total costs were £13.71/patient higher (95% confidence interval (CI): −3.19, 30.60) in the printed summary group than that in the no printed summary group (where only the Patient Update Information Sheet, informing patients how to access the webpage ± the email list, was mailed), but this difference was not statistically significant (p = 0.108). The differences in costs of the other comparisons were smaller and also not statistically significant. There was no evidence of interaction between interventions. Supplemental Table S7 shows the average costs to site per patient by each of the eight factorial combinations. The biggest component of the total costs was staff time to send out the printed information, taking, on average, 11 min/patient (range 0–35) in the no printed summary group compared with the 46 min/patient in the printed summary group (range 0–144). This translated into a £16.72 higher (95% CI: 5.43, 28.01) cost per patient for time spent posting information in the printed summary group compared to no printed summary (p = 0.005). The amount of time spent dealing with queries from patients was about 10 min per patient (range 0–70), translating into a £2.85/patient lower (95% CI: −13.06, 7.45) cost of dealing with queries in the enhanced webpage group compared to the basic webpage group (p = 0.583) and to £3.63/patient lower (95% CI: −13.92, 6.67) cost in the printed summary group compared to the no printed summary group (p = 0.480). Other costs made up a small proportion of the overall costs, around £0.61/patient higher (95% CI: 0.46, 0.76) in the printed summary group compared to the no printed summary group (p < 0.001). S8 Text of the Supplemental Material summarises the resources used by the clinical trials unit in implementing the Show RESPECT interventions.

**Table 4.** Resources used by sites to share results with patient participants, per participant.

Variable	Overall	Webpage			Printed summary			Email list		
	Mean (Std dev.)	Basic	Enhanced	Mean difference^a^ (95% CI) p-value	No printed summary	Printed summary	Mean difference^a^ (95% CI) p-value	No invitation	Invitation	Mean difference^a^ (95% CI) p-value
Costs										
Estimated cost of time spent posting all information, British Pound Sterling (GBP)	14.10 (19.08)	11.81 (19.35)	16.28 (19.03)	4.71 (−6.53, 15.96) 0.401	5.13 (4.65)	21.85 (23.22)	16.72 (5.43, 28.01) 0.005	15.59 (22.60)	12.54 (14.96)	−3.25 (−14.50, 7.99) 0.561
Estimated cost of time spent dealing with queries, GBP	8.00 (16.08)	9.38 (16.64)	6.69 (15.81)	−2.85 (−13.06, 7.45) 0.583	9.56 (17.02)	6.65 (15.49)	−3.63 (−13.92, 6.67) 0.480	7.80 (18.88)	8.20 (12.99)	0.45 (−9.80, 10.71) 0.929
Other costs, GBP	1.01 (0.38)	1.01 (0.36)	1.00 (0.41)	0.00 (−0.15, 0.15) 0.984	0.68 (0.27)	1.29 (0.19)	0.61 (0.46, 0.76) <0.001	1.04 (0.42)	0.98 (0.34)	−0.08 (−0.23, 0.07) 0.310
Total costs^b^, GBP	23.11 (27.00)	22.20 (32.73)	23.97 (20.93)	1.91 (−14, 18.74) 0.819	15.37 (17.13)	29.79 (32.18)	13.71 (−3.19, 30.60) 0.108	24.43 (33.71)	21.72 (18.29)	−2.87 (−19.70, 13.95) 0.731
Time										
Estimated hours sending out all printed information	0.49 (0.64)	0.36 (0.55)	0.61 (0.70)	0.26 (−0.11, 0.62) 0.161	0.18 (0.16)	0.76 (0.77)	0.59 (0.22, 0.96) 0.002	0.53 (0.73)	0.45 (0.55)	−0.08 (−0.44, 0.28) 0.657
Estimated hours dealing with queries	0.17 (0.27)	0.19 (0.29)	0.15 (0.25)	−0.04 (−0.21, 0.13) 0.631	0.21 (0.26)	0.14 (0.28)	−0.08 (−0.25, 0.09) 0.343	0.12 (0.23)	0.22 (0.30)	0.10 (−0.07, 0.26) 0.237
Other										
Approximate number of participants who had queries	0.34 (0.49)	0.44 (0.53)	0.25 (0.44)	−0.19 (−0.49, 0.11) 0.198	0.45 (0.58)	0.25 (0.38)	−0.23 (−0.52, 0.07) 0.131	0.33 (0.46)	0.35 (0.53)	0.03 (−0.27, 0.33) 0.840

CI: confidence interval.

a Adjusted for strata and other randomisations.

b Primary outcome for site staff data.

### Site staff views on the experience of sharing trial results with patients in Show RESPECT

#### Overall views of the process of sharing results in Show RESPECT

Site staff described their experiences of sharing results in positive terms, as ‘easy’, ‘not complex’, ‘achievable’, ‘not time-consuming’ and ‘working well’. They appreciated the ‘clear instructions’ provided.

#### Preparing patients to receive the trial results

The need to prepare patients to receive results was discussed repeatedly in site staff interviews and questionnaires. The importance of preparing patients stemmed partly from recognition that not all would want to know the results, and so it was important to offer the opportunity to opt in or out. Views differed on the timepoint at which this should be done, with some suggesting it should be done when patients join the trial. Others thought patients might change their minds between joining the trial and the results becoming available and so would need to be asked nearer the time:

I don’t believe in just sending information without first asking for their consent or asking them if they’re interested in doing this, because some do want to know and some don’t want to know. (BMRNI04: research burse, medium strata site)

Another driver behind the need to prepare patients prior to sharing results was that results may potentially be upsetting or confusing:

If the results are difficult to interpret or complex you may want to add something like the results are complex and you may want to go through it with your doctor, or something like that. (EBLMCLI02: oncologist, large and medium strata sites)

In Show RESPECT, the Patient Update Information Sheet was designed to prepare patients to receive results and to give them the opportunity to opt-out or access the interventions. Some site staff added an additional preliminary step, telephoning or talking to patients in clinic to inform them that the Patient Update Information Sheet was coming:

We sent the information sheets out, then contacted them and let them know they’re coming … That’s a really good way of doing it because if that had just ended up on their doorstep, they’d have read it and then just probably thrown it in the bin because it didn’t come with any compliment[s] slip or they didn’t really know what it was. (GMTCI02: trial coordinator, medium strata site)

However, not all site staff agreed that this step was needed or that it was a good idea:

I don’t agree with phoning the patients, just because, you know, a lot of our patients, you know, are busy with day-to-day life and it’s not, I don’t think it’s nice just calling them and reminding them of it all. (FLTCI01: trial coordinator, large strata site)

#### Further follow-up and support

There was recognition that some patients may need further support or have questions about the results. Some site staff phoned patients after they sent the Patient Update Information Sheet or printed summary to see if they had any questions or needed further support. Others included a note with the results inviting patients to make contact with any questions.

#### Concerns and challenges

Most site staff questionnaire respondents (88%, 60/68) reported having no concerns about how they shared the ICON8 results with patients, with no statistically significant differences between the randomised interventions. Similarly, around three quarters of site staff reported not finding anything challenging about sharing the ICON8 results, again with no statistically significant differences between the arms (Table 5). Some of the site staff were concerned about the emotional impact of sharing results: Some were uncomfortable sharing information on average progression-free survival times, while others were concerned that those who felt they had benefitted from trial participation may be upset to learn that the trial did not find a benefit overall.

**Table 5.** Site staff views and experience around sharing the ICON8 results with patient participants.

Variable	Overall	Webpage			Printed summary			Email list		
	n (%)	Basic, n (%)	Enhanced, n (%)	Odds ratio (95% CI) p-value	No printed summary, n (%)	Printed summary, n (%)	Odds ratio (95% CI) p-value	No invitation, n (%)	Invitation, n (%)	Odds ratio (95% CI) p-value
Any concerns about how you shared the results?				0.71 (0.14, 3.58) 0.679			2.59 (0.45, 14.83) 0.284			3.15 (0.56, 17.59) 0.191
Yes	8 (12)	5 (14)	3 (9)		2 (6)	6 (17)		2 (6)	6 (18)	
No	60 (88)	30 (86)	30 (91)		31 (94)	29 (83)		33 (94)	27 (82)	
Did you find anything challenging about sharing the results?				5.94 (0.80, 44.27) 0.082			3.31 (0.47, 23.52) 0.231			2.57 (0.40, 16.59) 0.321
No	52 (76)	30 (86)	22 (67)		27 (82)	25 (71)		29 (83)	23 (70)	
Yes	16 (24)	5 (14)	11 (33)		6 (18)	10 (29)		6 (17)	10 (30)	
How many participants contacted you with queries?^a^										
0	11 (27)	4 (17)	7 (39)	4.8 (0.53, 43.13) 0.161	3 (16)	8 (36)	0.76 (0.07, 8.33) 0.824	2 (13)	9 (36)	1.23 (0.14, 10.14) 0.851
1 to 2	24 (59)	17 (74)	7 (39)		12 (63)	12 (55)		11 (69)	13 (52)	
3 to 5	6 (15)	2 (9)	4 (22)		4 (21)	2 (9)		3 (19)	3 (12)	
How able did you feel to help with queries?^b^										
Very difficult	0 (0)	0 (0)	0 (0)	0.27 (0.02, 3.56) 0.317	0 (0)	0 (0)	2.61 (0.24, 28.40) 0.432	0 (0)	0 (0)	0.75 (0.08, 7.32) 0.802
Quite difficult	1 (3)	0 (0)	1(8)		1 (6)	0 (0)		0 (0)	1 (6)	
Not sure	4 (13)	2 (11)	2 (17)		3 (18)	1 (7)		0 (0)	4 (22)	
Quite easy	15 (48)	9 (47)	6 (50)		9 (53)	6 (43)		9 (69)	6 (33)	
Very easy	11 (35)	8 (42)	3 (25)		4 (24)	7 (50)		4 (31)	7 (39)	
Should the way you shared results with participants be the standard approach for other trials?										
No	16 (24)	6 (17)	10 (30)	0.39 (0.07, 2.42) 0.318	9 (27)	7 (20)	1.7 (0.32, 9.04) 0.532	8 (23)	8 (24)	0.84 (0.16, 4.45) 0.838
Yes	52 (76)	29 (83)	23 (70)		24 (73)	28 (80)		27 (77)	25 (76)	
Would you do anything different for future trials?										
No	52 (79)	28 (82)	24 (75)	1.65 (0.48, 5.67) 0.424	26 (81)	26 (76)	1.43 (0.41, 5.02) 0.573	28 (80)	24 (77)	1.06 (0.31, 3.62) 0.93
Yes	14 (21)	6 (18)	8 (25)		6 (19)	8 (24)		7 (20)	7 (23)	

CI: confidence interval.

a Ordinal odds ratio.

b For the ordinal regression analysis, the very and quite difficult and not sure categories were merged.

Oncologists expressed the need for care in how sharing results is done to ensure patients are not unnecessarily distressed or confused. Patient and public involvement in the preparation of information about results for patients was identified as a way of mitigating the risk of causing upset:

We do have a duty to give the patient information, it’s just being wise and careful to give that information well, in a way that patients can understand … And like any information we have to time that well and be sensible about who we’re giving that to. (EBLMCLI02: oncologist, large and medium strata sites)

The other main challenges identified by site staff were the time needed to share results via mailed information. This was particularly an issue for staff at sites with larger numbers of ICON8 patients. Staff at smaller sites recognised that although it may not have been a problem for them in this study, it would be challenging in studies with many patients at a site:

It was just so time-consuming to send, you know, the results out to so many patients. (HLRNI03: research nurse, large strata site)

If site staff had not had contact with a patient for a while and were aware the patient was unwell, this did raise some questions over whether they should send the results. Similarly, if patients had transferred between sites, it could cause confusion over who was responsible for sharing results with that patient. For some site staff, giving information out remotely, rather than face-to-face, was challenging as they could not gauge the reaction of patients.

#### Dealing with queries from patients

Just over a quarter of site staff questionnaire respondents reported that no patients contacted them with queries, while almost 60% were only contacted by one or two patients (Table 5). There were no significant differences between the randomised arms in the number of patient queries. Eighty-three percent of questionnaire respondents felt it was quite or very easy to deal with queries patients raised, with no significant between-arm differences.

#### Sharing results in future trials

Most site staff respondents said the way they had shared results with patients in Show RESPECT should be the standard approach for other trials (76%), with no significant differences between the randomised arms. Similarly, 79% said they would not do anything different for future trials (Table 5).

## Discussion

Providing results to patients in the form of opt-out printed summaries increased costs to sites of sharing results by around £14 per patient. Most of that increased cost was attributable to staff time in mailing printed information. These costs, and those incurred by the clinical trials unit, are small (1% or less) compared with the overall cost of trials, which previous research has estimated to be around £2987^27^ or £7890^28^ per participant on average, in the UK.

The processes and methods used in Show RESPECT to share results with patients were seen as both appropriate and feasible by site staff. Preparing patients to receive trial results was an important step in this process (similar to the findings of the BRACELET study).^29^ Some site staff viewed the Patient Update Information Sheet alone as sufficient, while others felt more comfortable talking to the patients first to let them know what to expect, and some felt it should have been covered in the informed consent process when patients joined the trial. Mailing out the printed information was generally reported to be easy and not too time-consuming although staff at sites with large numbers of ICON8 patient participants found it more burdensome. Site staff received few queries from patients about the results.

A key strength of these results is that they are from a mixed-methods randomised controlled study within a trial. The randomised design allows us to be confident that the differences we observed were due to the interventions rather than other factors, while the qualitative data allow us to explore the views of site staff on their experience of sharing trial results within the Show RESPECT study. The study included sites of different types and numbers of ICON8 patient participants, allowing us to transfer our results on feasibility and acceptability to other trials with similar site characteristics. However, Show RESPECT only looked at sharing the results of a single trial, and care needs to be taken when transferring the results to other trials in different diseases, health systems and results scenarios. Show RESPECT was not powered to detect differences in secondary outcome measures (which includes the outcomes reported in this article). This means there may have been real differences we were unable to detect. The questionnaires used to collect data on time spent on activities asked respondents to select the category that reflected the time they had spent, rather than giving precise figures. Similarly, our costs of time for different job roles at sites are based on a generic research costing tool, rather than the actual cost of each individual staff member. This means our cost figures are estimates rather than precise reflections of the time and cost of these activities. The resources required at the clinical trials unit level, reported in the S8 Text of the Supplemental Material, do not include the costs of translation, which should be included in future studies to ensure people who have different first languages are able to access the results.^30^ Another limitation is our cost estimates do not distinguish between fixed and per-patient costs. Economies of scale mean per-patient costs to the clinical trials units are likely to be lower in trials with more patients. There may also be economies of scale at the site level, meaning average costs to site per patient may be lower at larger sites and in larger trials.

Previously reported results from Show RESPECT showed that printed summaries improved patient satisfaction with how the results were shared and enabled more people who wanted to know the results to find them out.^7^ The results reported here show that this approach is feasible for site staff to implement and acceptable to them. This echoes findings from previous research, where study staff reported that disseminating trial results summaries was simple, straightforward and not time-consuming and that patient queries were not common and did not require substantial amounts of time to deal with.^31^

We believe this is the first study to provide detailed information about the costs of different approaches to sharing results with trial participants from both a site and clinical trials unit perspective. Our results include the human resource, printing and posting costs and therefore reflect a more accurate cost estimate. These costs should inform those planning results dissemination in future trials to ensure adequate resources are available (both budget and staff time) to share results with patients. Funders have a role to play in encouraging researchers to do this and in ensuring plans for sharing results are implemented. Further research is needed to determine the extent to which approaches to sharing results with trial participants need to differ for different populations, disease states, study questions and settings.

## Conclusions

We found that the process and communication approaches used within the Show RESPECT study for sharing results with patients were acceptable and feasible for site staff. The combination of a webpage and printed summary, which resulted in the highest participant satisfaction with how the results were shared, came at a modest cost that could be incorporated into trial budgets. The information on the process and resource requirements for the approaches used in Show RESPECT can guide others seeking to plan for sharing results.

## Supplemental Material

sj-docx-1-ctj-10.1177_17407745231186088 – Supplemental material for Site staff perspectives on communicating trial results to participants: Cost and feasibility results from the Show RESPECT cluster randomised, factorial, mixed-methods trial Supplemental material, sj-docx-1-ctj-10.1177_17407745231186088 for Site staff perspectives on communicating trial results to participants: Cost and feasibility results from the Show RESPECT cluster randomised, factorial, mixed-methods trial by Annabelle South, Julia Bailey, Barbara E Bierer, Eva Burnett, William J Cragg, Carlos Diaz-Montana, Katie Gillies, Talia Isaacs, Nalinie Joharatnam-Hogan, Claire Snowdon, Matthew R Sydes and Andrew J Copas in Clinical Trials

sj-docx-2-ctj-10.1177_17407745231186088 – Supplemental material for Site staff perspectives on communicating trial results to participants: Cost and feasibility results from the Show RESPECT cluster randomised, factorial, mixed-methods trial Supplemental material, sj-docx-2-ctj-10.1177_17407745231186088 for Site staff perspectives on communicating trial results to participants: Cost and feasibility results from the Show RESPECT cluster randomised, factorial, mixed-methods trial by Annabelle South, Julia Bailey, Barbara E Bierer, Eva Burnett, William J Cragg, Carlos Diaz-Montana, Katie Gillies, Talia Isaacs, Nalinie Joharatnam-Hogan, Claire Snowdon, Matthew R Sydes and Andrew J Copas in Clinical Trials

sj-docx-5-ctj-10.1177_17407745231186088 – Supplemental material for Site staff perspectives on communicating trial results to participants: Cost and feasibility results from the Show RESPECT cluster randomised, factorial, mixed-methods trial Supplemental material, sj-docx-5-ctj-10.1177_17407745231186088 for Site staff perspectives on communicating trial results to participants: Cost and feasibility results from the Show RESPECT cluster randomised, factorial, mixed-methods trial by Annabelle South, Julia Bailey, Barbara E Bierer, Eva Burnett, William J Cragg, Carlos Diaz-Montana, Katie Gillies, Talia Isaacs, Nalinie Joharatnam-Hogan, Claire Snowdon, Matthew R Sydes and Andrew J Copas in Clinical Trials

sj-docx-6-ctj-10.1177_17407745231186088 – Supplemental material for Site staff perspectives on communicating trial results to participants: Cost and feasibility results from the Show RESPECT cluster randomised, factorial, mixed-methods trial Supplemental material, sj-docx-6-ctj-10.1177_17407745231186088 for Site staff perspectives on communicating trial results to participants: Cost and feasibility results from the Show RESPECT cluster randomised, factorial, mixed-methods trial by Annabelle South, Julia Bailey, Barbara E Bierer, Eva Burnett, William J Cragg, Carlos Diaz-Montana, Katie Gillies, Talia Isaacs, Nalinie Joharatnam-Hogan, Claire Snowdon, Matthew R Sydes and Andrew J Copas in Clinical Trials

sj-docx-7-ctj-10.1177_17407745231186088 – Supplemental material for Site staff perspectives on communicating trial results to participants: Cost and feasibility results from the Show RESPECT cluster randomised, factorial, mixed-methods trial Supplemental material, sj-docx-7-ctj-10.1177_17407745231186088 for Site staff perspectives on communicating trial results to participants: Cost and feasibility results from the Show RESPECT cluster randomised, factorial, mixed-methods trial by Annabelle South, Julia Bailey, Barbara E Bierer, Eva Burnett, William J Cragg, Carlos Diaz-Montana, Katie Gillies, Talia Isaacs, Nalinie Joharatnam-Hogan, Claire Snowdon, Matthew R Sydes and Andrew J Copas in Clinical Trials

sj-docx-8-ctj-10.1177_17407745231186088 – Supplemental material for Site staff perspectives on communicating trial results to participants: Cost and feasibility results from the Show RESPECT cluster randomised, factorial, mixed-methods trial Supplemental material, sj-docx-8-ctj-10.1177_17407745231186088 for Site staff perspectives on communicating trial results to participants: Cost and feasibility results from the Show RESPECT cluster randomised, factorial, mixed-methods trial by Annabelle South, Julia Bailey, Barbara E Bierer, Eva Burnett, William J Cragg, Carlos Diaz-Montana, Katie Gillies, Talia Isaacs, Nalinie Joharatnam-Hogan, Claire Snowdon, Matthew R Sydes and Andrew J Copas in Clinical Trials

sj-pdf-3-ctj-10.1177_17407745231186088 – Supplemental material for Site staff perspectives on communicating trial results to participants: Cost and feasibility results from the Show RESPECT cluster randomised, factorial, mixed-methods trial Supplemental material, sj-pdf-3-ctj-10.1177_17407745231186088 for Site staff perspectives on communicating trial results to participants: Cost and feasibility results from the Show RESPECT cluster randomised, factorial, mixed-methods trial by Annabelle South, Julia Bailey, Barbara E Bierer, Eva Burnett, William J Cragg, Carlos Diaz-Montana, Katie Gillies, Talia Isaacs, Nalinie Joharatnam-Hogan, Claire Snowdon, Matthew R Sydes and Andrew J Copas in Clinical Trials

sj-pdf-4-ctj-10.1177_17407745231186088 – Supplemental material for Site staff perspectives on communicating trial results to participants: Cost and feasibility results from the Show RESPECT cluster randomised, factorial, mixed-methods trial Supplemental material, sj-pdf-4-ctj-10.1177_17407745231186088 for Site staff perspectives on communicating trial results to participants: Cost and feasibility results from the Show RESPECT cluster randomised, factorial, mixed-methods trial by Annabelle South, Julia Bailey, Barbara E Bierer, Eva Burnett, William J Cragg, Carlos Diaz-Montana, Katie Gillies, Talia Isaacs, Nalinie Joharatnam-Hogan, Claire Snowdon, Matthew R Sydes and Andrew J Copas in Clinical Trials
