# Supplementary material for: Site staff perspectives on communicating trial results to participants: Cost and feasibility results from the Show RESPECT cluster randomised, factorial, mixed-methods trial
Source: Clin Trials. 2023 Jul 29;20(6):649–60. doi: 10.1177/17407745231186088 (PMC10638850; doi:10.1177/17407745231186088)
Supplement: sj-docx-2-ctj-10.1177_17407745231186088 – Supplemental material for Site staff perspectives on communicating trial results to participants: Cost and feasibility results from the Show RESPECT cluster randomised, factorial, mixed-methods trial [file sj-docx-2-ctj-10.1177_17407745231186088.docx]

# S2 Text: Secondary outcomes collected from site staff

- Staff concerns with the interventions and process of communicating results;
- Time taken to deliver the Show RESPECT interventions;
- Proportion of respondents reporting challenges with implementing the Show RESPECT interventions;
- Number of queries received from patients following receipt of results;
- Time spent dealing with patient queries;
- Non-staff costs incurred by sites;
- Proportion of staff respondents saying the intervention(s) they were randomised to should become standard practice for the trials they are involved in;
- Proportion of respondents saying they would do something differently next time they shared results with participants;
- How easy respondents said they found dealing with patient queries about results;

## Secondary outcome from data collected from CTU staff

- Time and costs to the Clinical Trials Unit for developing, testing and distributing the interventions.
