# Supplementary material for: Site staff perspectives on communicating trial results to participants: Cost and feasibility results from the Show RESPECT cluster randomised, factorial, mixed-methods trial
Source: Clin Trials. 2023 Jul 29;20(6):649–60. doi: 10.1177/17407745231186088 (PMC10638850; doi:10.1177/17407745231186088)
Supplement: sj-docx-5-ctj-10.1177_17407745231186088 – Supplemental material for Site staff perspectives on communicating trial results to participants: Cost and feasibility results from the Show RESPECT cluster randomised, factorial, mixed-methods trial [file sj-docx-5-ctj-10.1177_17407745231186088.docx]

# S5 Text: Research team and reflexivity

## Personal characteristics

The interviews and analysis were carried out by AS. AS holds an MPhil and MSc, and this work forms part of her PhD. She is a research communication specialist, and is employed by a clinical trials unit to communicate trial results. She is female, and has been trained in qualitative research methods.

## Relationship with participants

She was known to site staff interviewees as the Chief Investigator of the Show RESPECT study, having led the online site training for the trial, and corresponding with sites about the study by email. She had not met participants in person prior to the interviews taking place. She introduced herself to participants as the Chief Investigator of the study, and the Policy, Communications & Research Impact Coordinator at the Clinical Trials Unit, but did not discuss her personal reasons for conducting the research, focusing instead on what is known from the literature about this topic, the research gap and the desire to generate evidence to inform practice. She did not inform participants that she had led the development of the Show RESPECT interventions. Her interest in this research topic comes from her professional experience of trying to communicate results to trial participants, but not all participants who want to learn results getting to find them out.

Prior to the study, AS believed that the Posted Printed Summary approach was likely to improve patient satisfaction with how the results were shared, but that there may be feasibility issues with implementing it. She expected the Enhanced Webpage to result in higher patient satisfaction than the Basic Webpage, and for there not to be differences in feasibility between the two webpages from a feasibility perspective. At the time of conducting the interviews, she was aware that no-one had signed up to the email list, so was expecting to hear that this approach was not useful for this patient population. She was also aware from site logs that sites reported sending the Patient Update Information Sheet and Printed Summaries to all applicable participants.
