# Supplementary material for: Site staff perspectives on communicating trial results to participants: Cost and feasibility results from the Show RESPECT cluster randomised, factorial, mixed-methods trial
Source: Clin Trials. 2023 Jul 29;20(6):649–60. doi: 10.1177/17407745231186088 (PMC10638850; doi:10.1177/17407745231186088)
Supplement: sj-docx-6-ctj-10.1177_17407745231186088 – Supplemental material for Site staff perspectives on communicating trial results to participants: Cost and feasibility results from the Show RESPECT cluster randomised, factorial, mixed-methods trial [file sj-docx-6-ctj-10.1177_17407745231186088.docx]

Show RESPECT: topic guide for interviews with ICON8 site staff

# Research questions *[for reference, not for asking participants]*

The aim of the study is to help us find the best ways to communicate results to people in research studies, and see what lessons we can learn from how this was done in ICON8.

1. What are the experiences and views of site staff in communicating the results of the ICON8 trial to the trial participants using the approaches tested in the Show RESPECT study, and how are these views shaped by their clinical setting or the interventions their site was randomised to?
2. What influences their views?
3. Which approaches to communicating trial results to participants are acceptable and feasible to implement for site staff?

# Introduction

- Thanks
- Who I am
- Go through participant info sheet
- Aim of study – to help us find the best ways to communicate results to people in research studies
  - Why I want to talk to you
  - Confidentiality and anonymity
  - Recording
  - Any questions?
- Go through consent form

**Start recording**

- Interested in your views and experiences - no right or wrong answers.
- We can stop at any time, or take a break, or skip questions you don’t want to answer

# About your role

- Please could you start by telling me your job title?
- What does that involve?
- What do you think are the most important aspects of your role?
  - *Do you have direct contact with participants?*
- What has been your role in the ICON8 study?
  - *How well do you know the participants?*
- What has been your role in the Show RESPECT study?
- How many trials do you work on?
- What diseases are the trials for?
- Do you find there are differences between the patients in the different trials in how much information they want?
  - Probe for details

# Information sources

- Do you generally find out the results of the trials you work on?
- How do you generally find out the results of trials you have worked on?

# Past experience of sharing trial results

- Is sharing overall trial results with participants something you do routinely?
- Do you have experience of sharing trial results with participants on previous trials? If so, please could you tell me about it?
  - *How?*
  - *When?*
  - *To whom?*
  - *Response*
    - *Has response varied b:*
      - *Trial arm*
      - *Results*
      - *Disease / stage*
  - *What support did you receive from*
    - *the CTU coordinating the trial?*
    - *Colleagues*

# Views about sharing results with trial participants in general

- What are your views about sharing results with trial participants in general?
  - *Do you think participants want to know?*
  - *Do you have any concerns about it in principle?*
    - *Any exceptions/ special cases?*
      - *What if the trial shows clear benefit from the intervention?*
      - *What if the trial shows no difference?*
      - *What if the trial shows harm from the intervention?*
      - *What if there is high mortality in the trial?*
  - *What do you think the benefits of sharing results might be?*
  - *What do you think the drawbacks of sharing results might be?*
  - *Do you think your views are shared by your colleagues?*

# Practicalities of sharing ICON8 results

- Could you talk me through the process you used for sharing the ICON8 results with participants?
  - *Time spent sending out the update sheet*
  - *Contacting participants – did you personalise the PUIS sheets / include a cover note / ring participants?*
  - *Timing*
  - *Time spent sending out the printed summary (if applicable)*
  - *Did you encounter any difficulties?*
- What would make it easier to share results with participants?

# ICON8 Participants' responses to finding out the results

- What responses have you had from participants to being offered the ICON8 results?
  - - *Questions?*

# Views on how the ICON8 results were shared

Your site was randomised to offer participants:

- What do you think about these methods to communicate the ICON8 results?
  - *Which method do you prefer? If so, why?*
  - *If the results had been difference, would that change which method you think should be used?*
    - *Showed clear benefit from weekly chemo*
    - *Showed harm or increased side-effects from weekly chemo?*
  - *Which method do you think your patients prefer? If so, why?*
  - *Are there some methods used which aren't good? If so, why?*
  - *Did you look at the webpage? If so, what did you think of it?*
    - *Look through webpage now*
      - *Figures*
      - *Further info & support contacts*
      - *Video*
      - *FAQ*
  - *How could it be improved?*
  - *Did you look at the printed summary? What did you think of it?*
    - *Look through printed summary now*
    - *What do you think of the content of the results section?*
  - *This is the email your participants could sign up to receive. What do you think of it?*

*If site was not randomised to printed summaries:*

- Do you know if any of your participants had difficulty accessing the webpage or email list?
  - - Did anyone ask you for a print out of the results?
    - Did anyone ask you to tell them the results?
  - What do you think of the process of informing participants of the results – sending the update information sheets first?
    - *Is that enough preparation for participants?*
    - *Do you think it is personal enough?*
    - *Is the opt-out approach for the printed summaries a good one?*

Some other sites were randomised to communicate results to participants using [*email list / basic webpage / enhanced webpage / printed summaries sent by post*]. *Show* *webpage/printed summary / email and give time to look at. Highlight differences from other webpage.*

- What do you think of this/ these approaches to communicating the ICON8 results?
  - *Content*
  - *Method of delivery*
  - *Which bits you like / dislike*
- Would you like to have been offered any of these approaches as well as or instead of the approaches you were able to offer to participants?
  - *Why?*

# Views on future practice

- How should the overall survival results of ICON8 be communicated to participants?
  - *Who should do it?*
  - *Which methods?*
  - *Which process?*
  - *To whom?*
    - *All patients*
    - *Relatives?*
  - *Why?*
  - *When?*

# General recommendations

- What do you think should be done to communicate the results of other trials to participants?
  - *Who should do it?*
  - *Methods?*
  - *Process?*
  - *To whom?*
    - *All patients*
    - *Relatives?*
  - *When?*
  - *Why?*
- Are there any exceptions to this?
  - *Does it matter which arm participants were on, in cases where there is a difference in outcomes?*
- What advice would you give to people working at sites on other trials on how to share the results with participants?
- What advice would you give to people working at clinical trials units on other trials on how to share the results with participants?
- Is there anything else you'd like to say about this topic?

# Thanks and wrap up

- Thank you
- This information will help us improve how we communicate results to trial participants
- We will share the overall results of this study with sites, when they are available.
