# Supplementary material for: Site staff perspectives on communicating trial results to participants: Cost and feasibility results from the Show RESPECT cluster randomised, factorial, mixed-methods trial
Source: Clin Trials. 2023 Jul 29;20(6):649–60. doi: 10.1177/17407745231186088 (PMC10638850; doi:10.1177/17407745231186088)
Supplement: sj-docx-7-ctj-10.1177_17407745231186088 – Supplemental material for Site staff perspectives on communicating trial results to participants: Cost and feasibility results from the Show RESPECT cluster randomised, factorial, mixed-methods trial [file sj-docx-7-ctj-10.1177_17407745231186088.docx]

# S7 Table: Total costs per participant, for each factorial combination of interventions

| **Interventions** | **Mean total cost per participant (GBP)** | **Standard Deviation** |
| --- | --- | --- |
| Control (Basic Webpage only) | 7.51 | 5.28 |
| Basic Webpage & Printed Summary | 42.05 | 58.36 |
| Basic Webpage & Email List Invitation | 15.76 | 16.96 |
| Basic Webpage & Printed Summary & Email List Invitation | 22.18 | 22.57 |
| Enhanced Webpage | 20.81 | 28.50 |
| Enhanced Webpage & Printed Summary | 26.86 | 23.05 |
| Enhanced Webpage & Email List Invitation | 17.47 | 12.08 |
| Enhanced Webpage & Printed Summary & Email List Invitation | 30.16 | 21.05 |
