# Supplementary material for: Site staff perspectives on communicating trial results to participants: Cost and feasibility results from the Show RESPECT cluster randomised, factorial, mixed-methods trial
Source: Clin Trials. 2023 Jul 29;20(6):649–60. doi: 10.1177/17407745231186088 (PMC10638850; doi:10.1177/17407745231186088)
Supplement: sj-docx-8-ctj-10.1177_17407745231186088 – Supplemental material for Site staff perspectives on communicating trial results to participants: Cost and feasibility results from the Show RESPECT cluster randomised, factorial, mixed-methods trial [file sj-docx-8-ctj-10.1177_17407745231186088.docx]

**Supplementary Text 8: Resources required from the Clinical Trials Unit (CTU)**

# Data collection

Data were collected from CTU staff who were members of the ICON8 or Show RESPECT study teams and were involved in developing or distributing the Show RESPECT interventions. MRC CTU trial team staff were asked to complete two short Show RESPECT CRFs. Data about the process of communicating results (first CRF) was collected immediately after interventions have been delivered. Data about the response from patients and sites (second CRF) was collected 2-3 months after administration of last intervention. One CRF set can be completed for each member of staff involved in dissemination of results. The CRF asked about the time they had spent on each of the Show RESPECT interventions. We also recorded other costs to the Unit, including printing and postage.

# Calculating the costs of staff time

The costs per hour of staff time were obtained for each staff member using the University’s research costing tool, Worktribe.

# Results

Developing, reviewing and implementing the Show RESPECT interventions took time from staff including a data manager, trial manager, programmer, statistician, communications specialist and clinical professor. The Email List was the most labour-intensive intervention for the CTU, taking around 41 hours of staff time, which cost approximately £1695. This was largely due to the time needed to develop and test the system to ensure that the details of any participants who signed up remained confidential. This was followed by the Patient Update Information Sheet, (36 hours, £1545) and Printed Summary (26.5 hours, £1182 total). The time and costs for the Enhanced Webpage (20.5 hours, £872 total) and Email Lists are underestimates, as both largely use the same text as the Printed Summary, but the time spent developing and reviewing this text is only counted in the Printed Summary row, to avoid double counting. The cost of the Basic Webpage was the lowest, although it also benefited from plain English text having already been developed for the printed summary (13.5 hours, £564 total).

Approximate time (hours) taken by CTU staff on developing, reviewing and disseminating the Show RESPECT interventions, and approximate cost of that time

|  | **Development time (hours)** | **Testing/ reviewing time (hours)** | **Distribution time (hours)** | **Total (hours)** | **Approximate cost of time (GBP)** |
| --- | --- | --- | --- | --- | --- |
| Patient Update information Sheet^[[1]](#footnote-1)^ | 17 | 9.5 | 9.5 | 36 | 1545 |
| Basic webpage | 4 | 9.5 | n/a | 13.5 | 564 |
| Enhanced webpage | 11 | 9.5 | n/a | 20.5 | 872 |
| Printed Summary | 11.5 | 13 | 2 | 26.5 | 1182 |
| Email list | 22 | 17 | 2 | 41 | 1695 |
| **Total** | **65.5** | **58.5** | **13.5** | **119.5** | **5858** |

In addition to the time involved in developing, testing and distributing the interventions, the CTU also incurred printing and postage costs for sending the Patient Update Information Sheet and Printed Summary to sites. The Patient Update Information Sheet was a 2-sided A4 document colour printed on high-quality paper using the CTU’s laser printers. The average cost to the CTU per participant for the Patient Update Information Sheet was £0.61. The Printed Summary was a 4-page A4 document professionally colour printed on 150gsm paper. The average cost to the CTU per participant for the Printed Summary was £0.69.

1. The Patient Update Information Sheet is how the links to the basic webpage, enhanced webpage, Email List were shared, along with opt-out information for the printed summary (ie. the other interventions were not stand-alone without the Patient Update Information Sheet). [↑](#footnote-ref-1)
