# Supplementary material for: Site staff perspectives on communicating trial results to participants: Cost and feasibility results from the Show RESPECT cluster randomised, factorial, mixed-methods trial
Source: Clin Trials. 2023 Jul 29;20(6):649–60. doi: 10.1177/17407745231186088 (PMC10638850; doi:10.1177/17407745231186088)
Supplement: sj-pdf-3-ctj-10.1177_17407745231186088 – Supplemental material for Site staff perspectives on communicating trial results to participants: Cost and feasibility results from the Show RESPECT cluster randomised, factorial, mixed-methods trial [file sj-pdf-3-ctj-10.1177_17407745231186088.pdf]

ICON8 Site Number: ☐☐☐☐   Site name…………………………………………………………………………..

Site Questionnaire Number: ☐☐

About this questionnaire

What is this for?
We would like to find out site staff experiences of sharing ICON8 trial results with the trial's participants. This is part of a wider project to find out the best way to share trial results with participants. Although most trial participants want to know results of research they take part in, often they are not told them, and there is not good evidence about the best method of getting results to participants.

When should it be completed?
You can complete this at any time after MRC CTU at UCL researchers have sent it to you.

Who should complete it?
You can complete it if you have been involved in disseminating ICON8 trial results to ICON8 participants. You should complete it to reflect your own views, not on behalf of your site. More than one person at each site can complete it, so if others at your site helped with disseminating ICON8 trial results, please do pass this questionnaire to them.

Do I have to complete it?
Completing this questionnaire is totally voluntary. If you do agree to provide your views, we would be very grateful, and you would be helping us improve how we share trial results with participants. Your responses will be treated in confidence, so please feel free to give honest answers.

How long will completing this take?
We estimate it will take about **5** minutes.

What will happen to the information provided on this questionnaire?
We will enter this data into a secure database, accessible only to those who need access. We will analyse the results to help us understand the main results of the project (the participants' perspectives). We will publish the results in a peer-reviewed journal, but nothing we publish will name individual participants, sites or staff members.

Where should I send the completed questionnaire?
When completed, please send to A. South, PO Box 75361, London, WC1A 9PL

ICON8 Site Number: ☐☐☐☐ Site name……………………………………………………………………………..

Site Questionnaire Number: ☐☐

Introduction

Please **only** complete this form if you were involved in the distribution of the Printed Summary and/or the Patient Update Information sheet to participants.

Please ask your site's main ICON8 trial contact to record that you have completed this questionnaire by entering your name next to the site questionnaire number, given above, on the **site feedback questionnaire log**. This will not be sent to MRC CTU at UCL, so your completion of this questionnaire will remain confidential.

1. What is today's date? d d / m m m / y y y y

Section 1—About you

This information will help us understand if different people have different views about how to share trial results.

2a. Which of the following most closely matches your current job role?

- ☐ Research nurse, research practitioner, research radiologist, clinical nurse specialist
- ☐ Clinician
- ☐ Clinical trial coordinator or research manager
- ☐ Data manager
- ☐ Trials administrator
- ☐ Other, b. please specify………………………………………………..

3. How long have you worked in clinical trials?

Less than 1 year	1 year to 5 years	6 to 10 years	Over 10 years
☐	☐	☐	☐

4. How many trials do you work on now?

1-5	6-10	Over 10
☐	☐	☐

ICON8 Site Number: ☐☐☐☐ Site name……………………………………………………………………………..

Site Questionnaire Number: ☐☐

5. Approximately how much of your time do you currently spend on ICON8?

Almost none	Around one day per week	Around two days per week	Three or more days per week
☐	☐	☐	☐

6. Approximately how long have you worked on the ICON8 trial?

Less than 1 year	1-2 years	3-4 years	5 or more years
☐	☐	☐	☐

7a. What has/will be your involvement been in sharing the ICON8 results? Tick all that apply.

- ☐ Sending the Printed Summary and/or the Patient Update Information sheet to patients by post
- ☐ Handling or answering queries from patients about the trial results
- ☐ Other, b. please specify ……………………………………………………………………

Section 2—Time and resource needed to send out results

8. Approximately how many hours did it take to send out the ICON8 Patient Update Information Sheet to all patients?

0-1	2-4	5-7	8-10	More than 10
☐	☐	☐	☐	☐

9. Approximately how many hours did it take to send out the Printed Summary to all patients?

0-1	2-4	5-7	8-10	More than 10
☐	☐	☐	☐	☐

10. What costs did your hospital incur for sending out the Printed Summary to participants, if any (excluding staff time)?

……
………...

ICON8 Site Number: ☐☐☐☐ Site name……………………………………………………………………………..

Site Questionnaire Number: ☐☐

Section 3—Your views

11a. Which do you think is the best method of sharing results with participants? Tick all that apply.

☐ Posted, Printed Summary

☐ Email

☐ Basic webpage

☐ Enhanced webpage (i.e. with videos and other extra content)

☐ Other, b. please specify…………………………………………………………………………

12. Why do you think this/these method(s) are the best…………………………………………… …..

……..

……...

13a. Do you have any concerns about how you shared the ICON8 results with patients?

Yes ☐ No ☐

13b. If so, please explain why……………………………………………………………………… …..

……..

14a. Was anything challenging about sharing the ICON8 results?

Yes ☐ No ☐

14b. If so, please explain:………………………………………………………………………………..

……...

……...

ICON8 Site Number: ☐☐☐☐ Site name……………………………………………………………………………..

Site Questionnaire Number: ☐☐

15a. Do you think the way(s) you shared results should be standard practice for trials you are involved in?

Yes ☐ No ☐

15b. If so, which method? And why? ……………………………………………………………………

……

………..

16a. Would you do anything differently for future trials whose results you are involved in communicating?

Yes ☐ No ☐

16b. If so, what would you do differently?..

……

………...

Thank you for completing this questionnaire.
Please return it to: A. South, PO Box 75361, London, WC1A 9PL

For office use only:

Date form received at CTU: dd - mmm - yyyy

Date form entered onto database : dd - mmm - yyyy

Initials of data enterer: ☐☐☐☐

Show RESPECT Site Feedback Questionnaire

Show Results to Participants Engaged in Clinical Trials

Form 3

ICON8 Site Number: ☐☐☐☐ Site name……………………………………………………………………………..

Site Questionnaire Number: ☐☐

About this questionnaire

What is this for?
We would like to find out site staff experiences of sharing ICON8 trial results with the trial's participants. This is part of a wider project to find out the best way to share trial results with participants. Although most trial participants want to know results of research they take part in, often they are not told them, and there is not good evidence about the best method of getting results to participants.

When should it be completed?
You can complete this at any time after MRC CTU at UCL researchers have sent it to you.

Who should complete it?
You can complete it if you have been involved in disseminating ICON8 trial results to ICON8 participants. You should complete it to reflect your own views, not on behalf of your site. More than one person at each site can complete it, so if others at your site helped with disseminating ICON8 trial results, please do pass this questionnaire to them.

Do I have to complete it?
Completing this questionnaire is totally voluntary. If you do agree to provide your views, we would be very grateful, and you would be helping us improve how we share trial results with participants. Your responses will be treated in confidence, so please feel free to give honest answers.

How long will completing this take?
We estimate it will take about **5** minutes.

What will happen to the information provided on this questionnaire?
We will enter this data into a secure database, accessible only to those who need access. We will analyse the results to help us understand the main results of the project (the participants' perspectives). We will publish the results in a peer-reviewed journal, but nothing we publish will name individual participants, sites or staff members.

Where should I send the completed questionnaire?
When completed, please send to A. South, PO Box 75361, London, WC1A 9PL

ICON8 Site Number: ☐☐☐☐ Site name……………………………………………………………………………..

Site Questionnaire Number: ☐☐

Introduction

Please **only** complete this form if you were involved in the handling or answering queries from patients about the trial results.

Please ask your site's main ICON8 trial contact to record that you have completed this questionnaire by entering your name next to the site questionnaire number, given above, on the **site feedback questionnaire log**. This will not be sent to MRC CTU at UCL, so your completion of this questionnaire will remain confidential.

1. What is today's date? d d / m m m / y y y y

Section 1—About you

This information will help us understand if different people have different views about how to share trial results.

2a. Which of the following most closely matches your current job role?

- ☐ Research nurse, research practitioner, research radiologist, clinical nurse specialist
- ☐ Clinician
- ☐ Clinical trial coordinator or research manager
- ☐ Data manager
- ☐ Trials administrator
- ☐ Other, b. please specify………………………………………………..

3. How long have you worked in clinical trials?

Less than 1 year	1 year to 5 years	6 to 10 years	Over 10 years
☐	☐	☐	☐

4. How many trials do you work on now?

1-5	6-10	Over 10
☐	☐	☐

ICON8 Site Number: ☐☐☐☐ Site name……………………………………………………………………………..

Site Questionnaire Number: ☐☐

5. Approximately how much of your time do you currently spend on ICON8?

Almost none	Around one day per week	Around two days per week	Three or more days per week
☐	☐	☐	☐

6. Approximately how long have you worked on the ICON8 trial?

Less than 1 year	1-2 years	3-4 years	5 or more years
☐	☐	☐	☐

7a. What has your involvement been in sharing the ICON8 results? Tick all that apply.

☐ Sending the Printed Summary or the Patient Update Information sheet to patients by post

☐ Handling or answering queries from patients about the trial results

☐ Other, b. please specify ……………………………………………………………………

Section 2—Participant responses

8. How many participants do you remember contacting you (by any means) with queries about the results?

1-2	3-5	5-10	More than 10
☐	☐	☐	☐

9. Approximately how many hours have you spent dealing with participant queries about the ICON8 trial results?

0-1	2-4	5-7	8-10	More than 10
☐	☐	☐	☐	☐

ICON8 Site Number: ☐☐☐☐ Site name……………………………………………………………………………..

Site Questionnaire Number: ☐☐

10. How able did you feel to help with participant queries?

It was very difficult to help	It was quite difficult to help	Not sure	It was quite easy to help	It was very easy to help
☐	☐	☐	☐	☐

11. Do you remember any participants being distressed or upset about the trial results?

Yes ☐ No ☐

Section 4—Your views

12a. Which do you think is the best method of sharing results with participants? Tick all that apply.

☐ Posted, Printed Summary

☐ Email

☐ Basic webpage

☐ Enhanced webpage (i.e. with videos and other extra content)

☐ Other, b. please specify……………………………………………..

13. Why do you think this/these method(s) are the best?

………………………………………………………………………………………………………..

………………………………………………………………………………………………………..

14a. Do you have any concerns about how you shared the ICON8 results with patients?

Yes ☐ No ☐

14b. If so, please explain why……………………………………………………………………...

………………………………………………………………………………………………………..

ICON8 Site Number: ☐☐☐☐ Site name…………………………………………………………………………..

Site Questionnaire Number: ☐☐

15a. Was anything challenging about sharing the ICON8 results?

Yes ☐ No ☐

15b. If so, please explain: ……………………………………………………………………………..

………...

16a. Do you think the way(s) you shared results should be standard practice for trials you are involved in?

Yes ☐ No ☐

16b. If so, which method? And why? ……………………………………………………………………

……….

………...

17a. Would you do anything differently for future trials whose results you are involved in communicating?

Yes ☐ No ☐

17b. If so, what would you do differently?...

……….

………...

Thank you for completing this questionnaire.
Please return it to: A. South, PO Box 75361, London, WC1A 9PL

For office use only:

Date form received at CTU: dd - mmm - yyyy

Date form entered onto database : dd - mmm - yyyy

Initials of data enterer: ☐☐☐☐
